# Continuous scanning for Bragg coherent X-ray imaging

**DOI:** 10.1038/s41598-020-69678-5

**Published:** 2020-07-29

**Authors:** Ni Li, Maxime Dupraz, Longfei Wu, Steven J. Leake, Andrea Resta, Jérôme Carnis, Stéphane Labat, Ehud Almog, Eugen Rabkin, Vincent Favre-Nicolin, Frédéric-Emmanuel Picca, Felisa Berenguer, Rim van de Poll, Jan P. Hofmann, Alina Vlad, Olivier Thomas, Yves Garreau, Alessandro Coati, Marie-Ingrid Richard

**Affiliations:** 1 CEA Grenoble, IRIG, MEM, NRS, Univ. Grenoble Alpes, 17 rue des Martyrs, 38000 Grenoble, France; 2 ESRF - The European Synchrotron, 71 Avenue des Martyrs, 38000 Grenoble, France; 3 CNRS, Université de Toulon, IM2NP UMR 7334, Aix Marseille Université, 13397 Marseille, France; 4 Synchrotron SOLEIL, L’Orme des Merisiers, Saint-Aubin, BP48, 91192 Gif-sur-Yvette, France; 5 Deutsches Elektronen-Synchrotron (DESY), Notkestraße 85, 22607 Hamburg, Germany; 6 Department of Materials Science and Engineering, Technion-Israel Institute of Technology, 3200003 Haifa, Israel; 7 Laboratory for Inorganic Materials and Catalysis, Department of Chemical Engineering and Chemistry, Eindhoven University of Technology, P.O. Box 513, 5600 MB Eindhoven, The Netherlands; 8 Laboratoire Matériaux et Phénomènes Quantiques, CNRS, UMR 7162, Université de Paris, 75013 Paris, France

**Keywords:** Condensed-matter physics, Materials for energy and catalysis, Imaging techniques

## Abstract

We explore the use of continuous scanning during data acquisition for Bragg coherent diffraction imaging, i.e., where the sample is in continuous motion. The fidelity of continuous scanning Bragg coherent diffraction imaging is demonstrated on a single Pt nanoparticle in a flow reactor at $400\,^\circ \hbox {C}$ in an Ar-based gas flowed at 50 ml/min. We show a reduction of 30% in total scan time compared to conventional step-by-step scanning. The reconstructed Bragg electron density, phase, displacement and strain fields are in excellent agreement with the results obtained from conventional step-by-step scanning. Continuous scanning will allow to minimise sample instability under the beam and will become increasingly important at diffraction-limited storage ring light sources.

## Introduction

Coherent diffraction imaging (CDI) is a lensless imaging technique, which exploits the coherent properties of a light source^1–5^. It allows one to reconstruct isolated two- or three-dimensional (2D or 3D) objects from their measured diffraction pattern using computational inversion algorithms to determine the phase of the scattered wave, which is not directly measured by the detector. The technique has been successfully applied in the Bragg geometry to recover the displacement and strain fields within finite crystals^6–10^. Bragg CDI (BCDI) has been recently used to image in situ and *operando* the structural evolution of crystals under external stimuli such as during nanoindentation^11^ or chemical reaction^12,13^.

In BCDI, one records the 3D intensity distribution around a Bragg peak by varying the incident angle or the sample azimuth of the X-ray beam with respect to the sample on the order of a few degrees (for instance, $\sim \pm 1^\circ$). However, its nature as a scanning technique poses strict requirements on the instrumentation. Typically, step-by-step scanning is used to record the 3D intensity. It has the drawback that the movement of the motor(s) and the acquisition are done in separate steps. In a conventional step-by-step scan, a large fraction of the scan time is spent waiting for motors to move as well as delays imposed by software and detector read out, the so-called ‘overhead’. Continuous scanning eliminates the overhead of the step-by-step scan^14–16^. There have been examples of continuous scanning on extended samples in the field of ptychography (CDI on samples larger than the beam), where the sample is spatially scanned with a focused coherent X-ray beam impinging upon it^17–19^. But, no demonstration of continuous scanning has been performed for BCDI on single objects. This method is particularly attractive for *operando* measurements soon to be realised at almost diffraction-limited X-ray sources. The increase in coherent flux will lead to shorter exposure times, therefore overheads in-between exposures will dominate the overall experiment time. Those should be minimised where possible.

Here, we aim to analyse the fidelity of continuous *versus* step-by-step scanning modes for BCDI measurements. We demonstrate continuous scanning BCDI on a selected Pt nanoparticle. We compare the BCDI measurement and reconstruction of the shape, phase, displacement and strain fields of the particle measured by conventional step-by-step and continuous scanning. The results obtained by both techniques are in excellent agreement. The reduction in the data acquisition time predestines continuous scanning BCDI for in situ and *operando* studies of functional materials. Finally, we go further and discuss the technical and analysis limitations that result from such data acquisition.

## Results

Three-dimensional (3D) diffraction data were collected as rocking curves of the rotation angle around the normal of the sample (here, $\mu$-angle—see Supplementary Figure S1) either in step-by-step or continuous scanning modes. Table 1 summarises the scanning conditions used for the two modes. In the step-by-step mode, the data was collected in steps of $0.0085^\circ$ in $\mu$-angle with a total of 128 frames and with a typical counting time of 3 s per angle. In the continuous scanning mode, the $\mu$-angle of the sample was scanned at a constant velocity ($0.00085^\circ$/0.3 s), while the same 2D photon-counting detector was measuring the data. In total, 1280 frames were collected. The motor movement and detector reading were performed simultaneously and synchronously by triggers from a reference clock, the detector dead time being negligible with a value of 290 ms^14^. The increase in frames by a factor 10 and decrease in exposure by the same factor means that the sample sees the same number of total photons in principle, if a fast shutter is installed to protect the sample in-between acquisitions. Thus, the two datasets should be completely equivalent. Note that if the beamline does not have a fast shutter (here, no fast shutter has been used), the sample will be exposed to more dose and possibly to beam damage during step-by-step compared to continuous scanning. The continuous scan was executed in 6 min 42 s compared to the step-by-step scan, which lasted 9 min 24 s (see Table 1). The overall time was then significantly reduced with a 30% gain, improving the reliability of data acquisition by limiting the time available for sample drift. For the data acquired during continuous scanning, we applied a binning factor of 10 along the rocking direction to match the conditions of the step-by-step data.

**Table 1:** Comparison between the two modes.

Mode	Step-by-step	Continuous scanning
Exposure time per points (s)	3	0.3
Number of points	128	1,280
Integrated intensity (counts)	16,950,077	17,127,507
Maximum intensity per second (counts/s)	31,587	30,817
Scan duration (s)	564	402
Overhead (s)	180	18

Characteristics of the step-by-step and continuous scanning modes.

**Figure 1:**
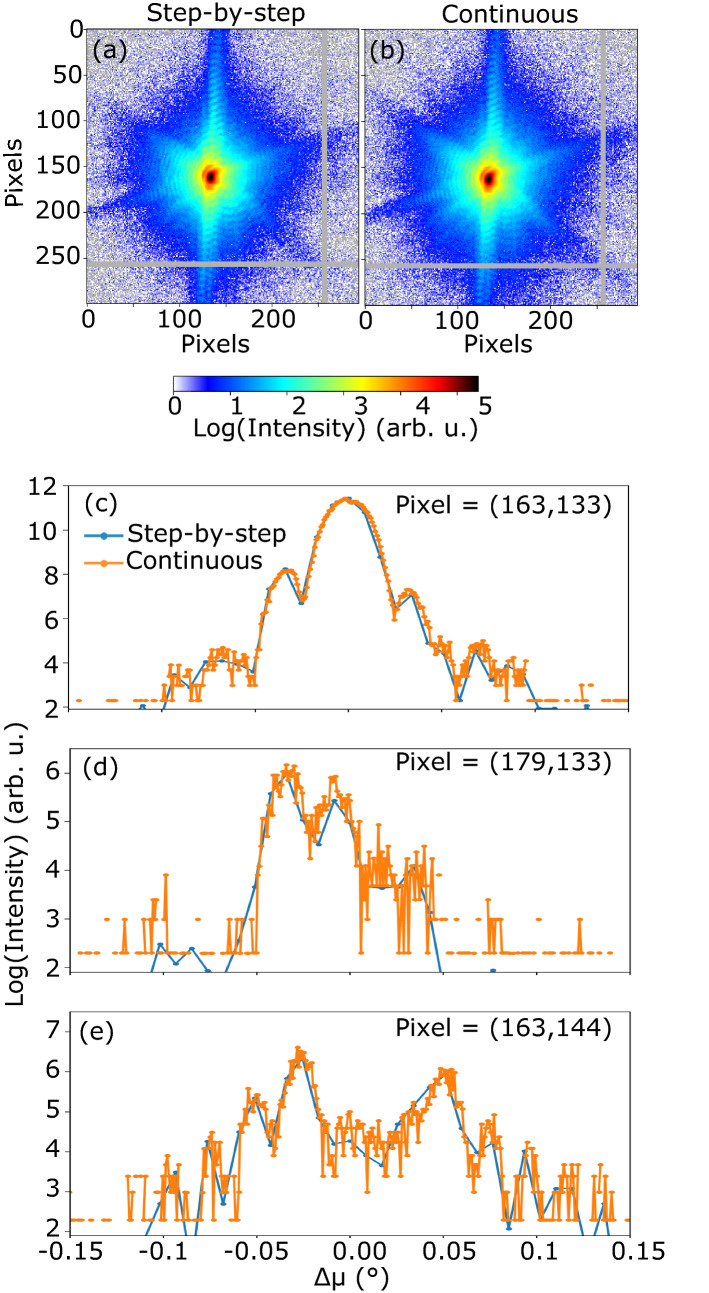
Comparison of intensity. Sum along the rocking direction of all the detector images acquired in the vicinity of the ${{\bar{\mathbf{1}}{} \mathbf{11}}}$ Pt reflection during (**a**) step-by-step and (**b**) continuous scanning for the same Pt particle. The data is cropped on a region of interest of the detector centered on the diffraction pattern. The grey area correspond to the detector gaps. Evolution of the measured intensity during step-by-step and continuous scanning (with a scaling factor of 10 for the intensity measured during continuous scanning) as a function of the rocking-angle and at different pixels of the 2D detector: (**c**) at pixel = (163, 133)—which corresponds to the maximum of the intensity, (**d**) at pixel = (179, 133) and (**e**) at pixel = (163, 144).

Figure 1a,b display the sum of all the detector images acquired in the vicinity of the ${{\bar{\mathbf{1}}{} \mathbf{11}}}$ Pt reflection for the same Pt particle, for the same angular range and for both methods (step-by-step and continuous scanning). The two patterns are very similar, which is also in agreement with the closely related values of the integrated intensity measured during step-by-step and continuous scanning (see Table 1). Note that the value of the maximum intensity measured for both techniques is also very close within 2.5% (see Table 1). The two patterns show facet streaks and well-defined fringes, which are the signature of a weakly strained particle. We have also calculated the Pearson correlation coefficient between the two 2D patterns shown in Fig. 1. It is equal to 0.983 for the full rocking curve, which demonstrates a very good correlation between the measurements performed using step-by-step and continuous scanning modes. Figure 1c–e display the measured intensity during step-by-step and continuous scanning as a function of the rocking-angle and at different pixel positions of the 2D detector. A scaling factor of 10 has been applied to the intensity measured during continuous scanning as the counting time per point is ten times smaller. We observe a good agreement between the two scanning methods as well as higher noise for data collected during continuous scanning.

### Comparison between the retrieved crystals with the two scanning modes

**Figure 2:**
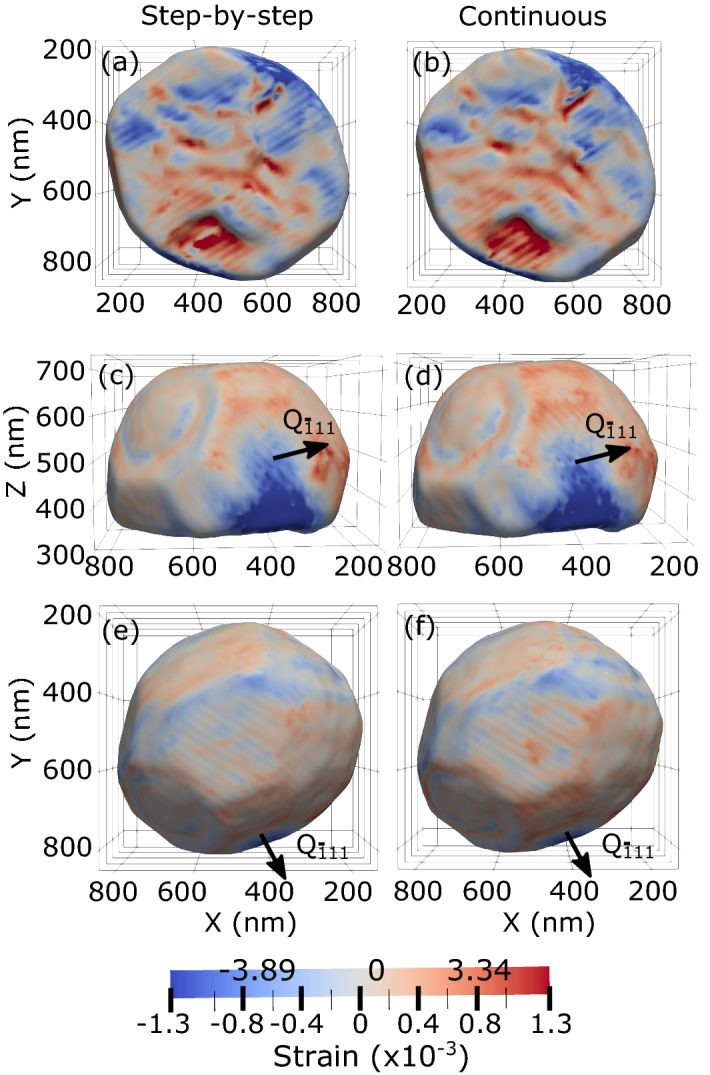
Comparison of the retrieved surface strain in 3D. Bottom (**a**,**b**), side (**c**,**d**) and top (**e**,**f**) views of the BCDI reconstruction of strain field along the [${{\bar{1}}11}$] direction, $\varepsilon _{{\bar{1}}11}$, drawn at 30% of the maximum Bragg electron density of the Pt nanoparticle measured by conventional step-by-step (**a**,**c**,**e**) and continuous (**b**,**d**,**f**) scanning. The direction of the scattering vector, ${\mathbf{Q}_{{\bar{\mathbf{1}}}{} \mathbf{11}}}$, is indicated in the figure.

The Bragg electron density and the phase were reconstructed from both diffraction patterns in the crystal frame (*X*, *Y* and *Z* being along the incident beam (downstream), outboard and vertically upward, respectively—see Figure S1). In Bragg geometry, the retrieved image is a complex field encoding the electronic density (in its modulus) and the displacement field $\vec {u}(\vec {r})$ projected onto the scattering vector (in its phase). As the ${{\bar{\mathbf{1}}{} \mathbf{11}}}$ Pt reflection has been measured, the retrieved displacement field is $u_{{\bar{1}}11}$ along the [${\bar{1}}$11] direction. The strain component is then derived from the retrieved displacement field: $\varepsilon _{{\bar{1}}11} = \frac{\partial u_{{\bar{1}}11}}{\partial y'}$, where $y'$ is along the [${\bar{1}}$11] direction. Note that it is more relevant to compare the strain instead of the phase or the displacement, since these are retrieved with an arbitrary constant, leading to an unknown offset in the reconstructed phase and displacement. Figure 2 displays different 3D views of the BCDI reconstruction of the strain field along the [${{\bar{1}}11}$] direction, $\varepsilon _{{\bar{1}}11}$, drawn at 30% of the maximum Bragg electron density of the Pt nanoparticle measured by both scanning methods. The 3D BCDI reconstruction of the phase and of the displacement field, $u_{{\bar{1}}11}$, is displayed in Figure S2. As shown in Figures 2 and S2, the reconstructed shape of the Pt crystal is a well faceted particle. The particle size is $\sim \,615$ (width) $\times \,640$ (length) $\times \,420$ (height) $\hbox {nm}^3$. The 3D BCDI reconstructions demonstrate that the reconstructed Bragg electron density, phase, displacement and strain fields are essentially the same for step-by-step and continuous scanning modes. For the retrieved strain component ($\varepsilon _{{\bar{1}}11}$), red corresponds to positive/tensile strain, while blue corresponds to negative/compressive strain along the [${\bar{1}}$11] direction. Interestingly, edges and facets of the crystal display opposite strain. For the displacement field (see Figure S2), edges of the crystal display negative displacement, while facets show preferentially positive displacement along the [${\bar{1}}$11].

**Figure 3:**
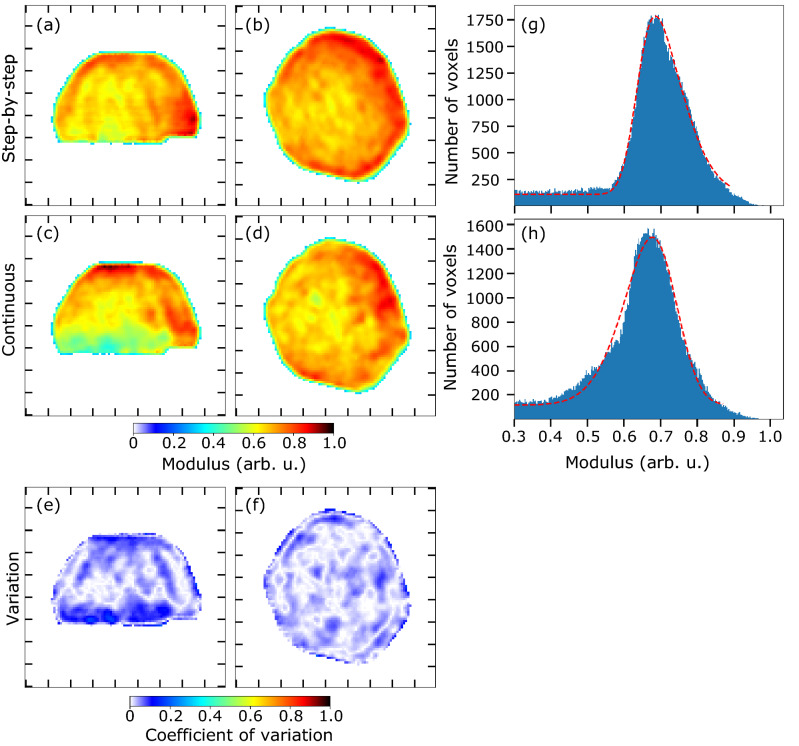
Comparison of retrieved modulus. (**a**,**b**) Central slice of the reconstructed modulus in *yz* and *xy* planes for the step-by-step scan. (**c**,**d**) Central slice of the reconstructed modulus in *yz* and *xy* planes for the continuous scan. (**e**,**f**) Coefficient of variation of the central slice of the reconstructed modulus in *yz* and *xy* planes. Ticks correspond to 100 nm. (**g**,**h**) Histograms of the reconstructed modulus for the step-by-step and continuous scanning modes with their corresponding fit (red curves).

We now analyse the reconstructed Bragg electron density or modulus of the crystal measured by step-by-step and continuous scanning modes in more detail. We considered partial coherence in the phase retrieval algorithm (see the phase retrieval section in Methods for more details). Figure 3 displays the reconstructed modulus in *yz* and *xy* planes for the step-by-step (a–b) and continuous (c–d) scans. The reconstructed modulus obtained by continuous scanning BCDI appears less homogeneous. The coefficient of variation (ratio of the standard deviation to the mean) of the reconstructed moduli in *yz* and *xy* planes is displayed in Fig. 3e,f. The coefficient of variation of the reconstructed moduli is defined by: $s = \sigma (m_1,m_2)/\mu (m_1,m_2)$, where $\sigma$ and $\mu$ are the standard deviation and the mean of the reconstructed moduli for the step-by-step ($m_1$) and continuous ($m_2$) scanning modes. Variations of up to 20% between the two methods are observed in some areas of the reconstructed modulus. Figure 3g,h show the histograms of the reconstructed modulus for the step-by-step and continuous scanning modes. The histogram of the modulus for the continuous scanning method shows a small shoulder on the left side of the intense peak, in agreement with the fact that the reconstructed modulus obtained by continuous scanning BCDI is less homogeneous. Nevertheless, both histograms show a sharp distribution (which is the signature of satisfactory reconstructions) of the modulus with a standard deviation of $0.11\pm 0.01$ for both methods.

**Figure 4:**
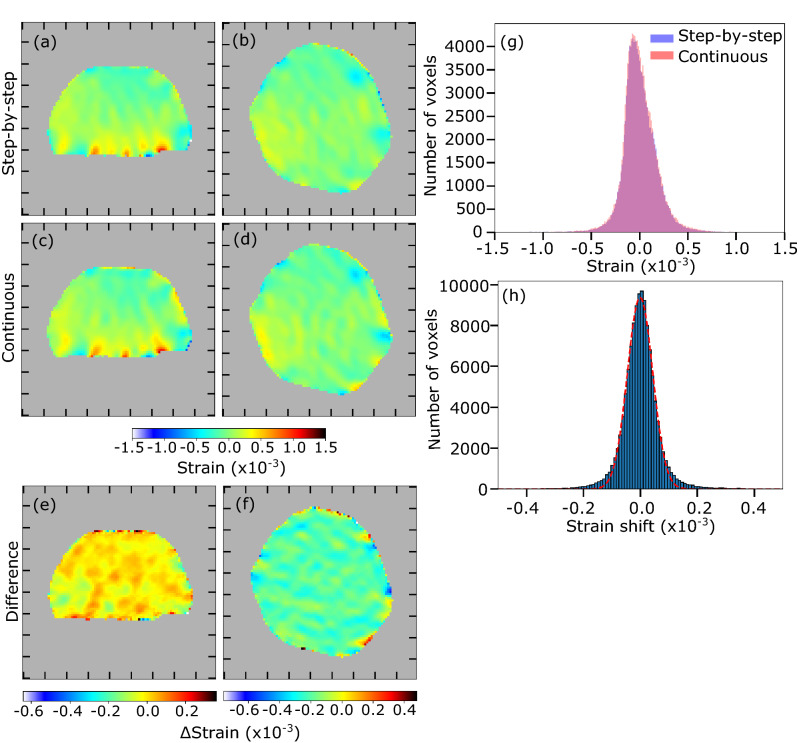
Quantitative comparison of retrieved strains. (**a**,**b**) Central slice of the reconstructed strain in *yz* and *xy* planes for the step-by-step scan, drawn at 30% of the reconstructed modulus. (**c**,**d**) Central slice of the reconstructed strain in *yz* and *xy* planes for the continuous scan, drawn at 30% of the reconstructed modulus. (**e**,**f**) Central slice of the difference of the reconstructed strains in *yz* and *xy* planes, drawn at 30% of the reconstructed modulus of the step-by-step scan. Ticks correspond to 100 nm. The colorbar in the difference maps reflects the full range of the slice only, not of the full crystal. (**g**) Histograms of the strain ($\varepsilon _{{\bar{1}}11}$) reconstructed for both scanning methods. (**h**) Histogram of the strain difference between the two scanning methods on a voxel-by-voxel basis as well as its Gaussian fit.

We then compared quantitatively the strain along the [${\bar{1}}$11] direction, $\varepsilon _{{\bar{1}}11}$, retrieved from the measurements of both scanning methods. Figure 4 displays the reconstructed strain, $\varepsilon _{{\bar{1}}11}$, in *yz* and *xy* planes for the step-by-step (a–b) and continuous (c–d) scans. As shown by the weak values of the difference of the reconstructed strains (see Fig. 4e,f), the strain retrieved by the two methods are in very good agreement. The same conclusion can be drawn from the reconstructed phase in *yz* and *xy* planes shown in Figure S3 for the step-by-step and continuous scans. Interestingly, in Fig. 4a,c, localised strain is observed at the crystal/substrate interface; it may arise from a dislocation network at the interface.

Histograms of the $\varepsilon _{{\bar{1}}11}$ strain for both methods are displayed in Fig. 4g. The two histograms are almost superimposed, indicating that both reconstructions are in very good agreement. We have also quantified the difference of the strain reconstructed for both scanning methods on a voxel-by-voxel basis, the voxel size being equal to $9.76^3\, \hbox {nm}^3$ for both reconstructions. The histogram shown in Fig. 4h illustrates the dispersion of the differences in the reconstructed strain values and follows a Gaussian behaviour. The standard deviation is $6\,\times \,10^{-5}$, which is within the accuracy of the technique^20^. We also compared quantitatively the [${\bar{1}}$11] displacement ($u_{{\bar{1}}11}$) reconstructed for both scanning methods (see Figure S3 of Supplementary materials). For the dispersion of the differences in the reconstructed phase values, the standard deviation is $7.18\,\times \,10^{-2}$ rad, corresponding to a small displacement of 2.6 pm, very close to the accuracy of the technique of 1 pm (this value from literature being better as multiple reflections have been used^21^). All these quantitative comparisons of the reconstructed strain and displacement demonstrate the very good agreement of the reconstructions from the step-by-step and continuous scanning methods. A better agreement is observed for the retrieved phase (and thus displacement and strain fields) compared to the retrieved modulus. This is consistent with the fact that the reconstruction of the phase is more robust than the one of the modulus, which often shows large fluctuations^22^.

### Impact on the spatial resolution

**Figure 5:**
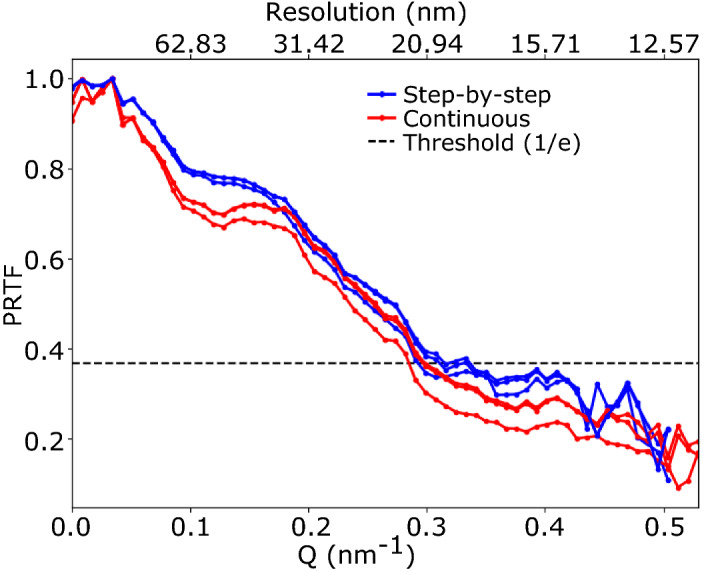
Spatial resolution. Estimation of the spatial resolution using phase retrieval transfer function (PRTF) for three sets of reconstructions obtained from the step-by-step and continuous scanning modes.

To assess the quality of the reconstructions, the spatial resolution of the reconstructed particle has been evaluated using the Phase Retrieval Transfer Function (PRTF)^23^. The cut-off value has been fixed at 1/*e*. As explained in Methods, to ensure the best reconstruction possible, we selected only the best 50 solutions (with lowest free log-likelihood^24^) out of 500 with random phase start. The free log-likelihood indicator is used as a metric evaluation of the solutions and allows to discriminate between them (see Figure S4). The final solution is then obtained through an eigen-decomposition of the best solutions and corresponds to the first mode of this decomposition. To evaluate the number of best solutions to keep for the eigenvalue decomposition, we have evaluated the spatial resolution as a function of the number of solutions. As shown by the PRTF curves in Figure S5, the resolution goes down with the number of solutions and then reaches a plateau for 30 solutions and more, where the PRTF curves give the same spatial resolution for a cut-off value of 1/*e*. In the following, we give the resolution obtained after keeping the best 50 reconstructions. Note that the PRTF is not a measure of the absolute resolution but of the consistency of the reconstructions. Nevertheless, it is often used to give an estimation of the resolution^23,25^. Figure 5 displays the PRTF for reconstructions obtained from the step-by-step and continuous scanning modes for three sets of solutions (i.e. three different runs of 500 reconstructions). We obtain a spatial resolution in the range of 20–22 nm for step-by-step and continuous scanning methods. Even if the resolution looks slightly higher for continuous scanning, the difference is negligible relative to the pixel size.

Finally, we have tested the impact of binning along the rocking direction on the spatial resolution of the reconstructed crystal measured by continuous scanning BCDI. Previously, for the data acquired during continuous scanning, we have applied a binning factor of 10 along the rocking direction to match the conditions (i.e., the oversampling) of the step-by-step data. Figure S6 estimates the spatial resolution using the PRTF for different binning factors along the rocking direction (from 1, i.e. no binning, to 20) of the data measured by continuous scanning. All the curves have been obtained from the first eigen-solution obtained by computing an eigenvalue decomposition over the 50 best reconstructions out of 500, like before. For all the binning conditions, the first eigen-solution shows a relative weight larger than 96%. The weight is an indicator of the correlation between solutions, ideally the relative weight of the first (strongest) eigen-solution should be as close to 100% as possible^24^. As shown in Figure S6, a good resolution is obtained for binning factors ranging from 2 to 20, even if binning smooths out the data. A poorer resolution is obtained for no binning. This may be explained by the reduced number of photons in each frame when no binning is applied. Interestingly, a binning factor of 20, which corresponds to an oversampling ratio, $\sigma$, of $\sim \,1.1$ along the rocking direction, still gives a good resolution. Good spatially-resolved reconstructions are then demonstrated with an oversampling ratio as low as 1.1 in the rocking direction. This value is lower than the oversampling criterion of $\sigma > 2^{1/3}$ in each direction, which is required to solve the phase problem^26^. Nevertheless, the overall 3D oversampling ratio of the data ($1.1\,\times \,7\,\times \,6 = 46.2$) is larger than 30, which has been determined as the low limit to achieve a reconstruction with quality^27^, and the fringes are not along the binning direction. These can explain why the reconstruction works even if $\sigma < 2^{1/3}$ in the binning direction. We also observed that for the step-by-step data, reconstruction works for a binning factor of 2 along the rocking direction. This corresponds to an oversampling of $\sim 1.1$ as for the data measured by continuous scanning. It is often needed to resample the raw data afterwards for increasing photon statistics or reducing the array size for more efficient data processing. Here, we binned the data measured by continuous scanning to match the conditions of the step-by-step data. It is also possible to reduce the array size by skipping data points^28^. As the continuous scanning mode generates more data, resampling may be applied and as demonstrated here, applying a binning factor up to 20 in this case does not degrade the spatial resolution of the reconstruction and allows faster data processing (reduced size of the dataset).

## Discussion

The two approaches used in the present work (step-by-step and continuous scanning methods) are very robust and give essentially the same results. This demonstrates that BCDI scans of a continuously moving sample can be reconstructed with high fidelity. The continuous scanning approach offers a faster measurement and the scanning time was significantly reduced with a 30% gain. This gain is significant, it can improve the reliability of the data acquisition, as there is less time for instabilities in the experiment to mitigate the results (e.g. sample or beam drifts). Sample instability is a bottleneck especially for in situ BCDI experiments at third generation synchrotron sources and diffraction-limited storage rings^29^. It also offers opportunities for new methods for sample characterisation. One could envisage running the scan 10 times at a further 10 reduction in exposure, this would negate the gains in overhead but the data quality would be improved. The typical method of cross correlation for data combination could be applied and those which were compromised due to instability removed, thus improving the efficiency of the photons and the total dose the sample receives. There is also an opportunity here for the identification of the onset of processes in *operando* measurements, typically a full 3D description of the Bragg peak is needed to confirm if the strain state or lattice parameter has changed, and this provides it, and could be exploited further using chrono-CDI type approaches^30^. Faster measurements are obviously important for studying time-dependent processes and thus increasing the time resolution of BCDI. An exposure time of 300 ms and a motor speed of $\sim \,0.0028^\circ$/s have been used here. At the SixS beamline at the SOLEIL synchrotron, faster measurements down to an exposure time of 50 ms and up to a maximum speed of ${4}^{\circ}\!/{\mathrm{s}}$ for the $\mu$ motor are possible. A compromise between speed and measured photons/flux should be made to preserve the reconstruction quality. This will be helped by the upgrade of the synchrotron sources, which will increase the coherent flux and make continuous scanning measurements faster.

To sum-up, continuous scanning BCDI was demonstrated on a selected Pt particle in a flow reactor. It gives essentially the same results as the conventional step-by-step scanning method. Conventional phase retrieval algorithms were used to reconstruct the Bragg electron density, phase and strain field of the particle. These results reconstructed from measurements using both step-by-step and continuous scanning modes are in good agreement, even for a continuously moving sample. Shifts of the phase field of $7.18\,\times \,10^{-2}$ rad that translates to a displacement as low as 2.5 pm were obtained for both scanning methods. The standard deviation of the differences in the reconstructed strain values is $6\,\times \,10^{-5}$, which is in the accuracy of the technique. Continuous scanning allows to minimise sample instability under the beam and will become increasingly important in the near future to benefit from the multiple upgrade projects currently being carried out or planned at several third-generation sources.

## Methods

### Sample growth

Pt nanocrystals were prepared by the solid-state dewetting of a 30-nm thin Pt film for 24 h at $1100\,^\circ \hbox {C}$ in air. The Pt film was deposited on $\alpha$-$\hbox {Al}_2 \hbox {O}_3$ (sapphire) with an electron beam evaporator. The Pt nanocrystals have their *c*-axis oriented along the [111] direction normal to the (0001) sapphire substrate.

### Experimental measurements

The BCDI experiment was performed at the SixS (Surface Interface X-ray Scattering) beamline of synchrotron SOLEIL, France (see Figure S1). The SixS beamline is dedicated to the study of X-ray scattering from surfaces and interfaces of hard and soft matter in various environments in the 5–20 keV energy range. The required beam size was obtained with a Fresnel zone-plate (focal distance of 20 cm), which focused the beam down to $\sim \,{3}\,\mu \hbox {m}$ (horizontally) $\times \, {2}\,\mu \hbox {m}$ (vertically). A coherent portion of the beam was selected with high precision slits by matching their horizontal and vertical gaps with the transverse coherence lengths of the beamline: ${20}\,\mu \hbox {m}$ (horizontally) and ${100}\,\mu \hbox {m}$ (vertically). A circular beam-stop, and a circular order-sorting aperture, were used to block the transmitted beam, and higher diffraction orders, respectively. The BCDI experiment was performed at a beam energy of 8.5 keV (wavelength of 1.46 Å). The sample was mounted in a dedicated reactor with the substrate surface oriented in the horizontal plane on a hexapod that was mounted on a 6-circle *z*-axis diffractometer. The study has been performed in grazing incidence geometry. The incident angle was fixed to $3^\circ$ and the asymmetric ${{\bar{\mathbf{1}}{} \mathbf{11}}}$ Pt reflection was measured. The diffracted beam was recorded with a 2D MAXIPIX photon-counting detector ($516\,\times \,516$ pixels with pixel size of ${55\,\mu \hbox {m}\,\times \,55\,\mu \hbox {m}}$) positioned on the detector arm at a distance of 1.22 m^31^. The in-plane ($\gamma$) and out-of-plane ($\delta$) angles of the detector were $35.7^\circ$ and $10.2^\circ$, respectively. Three-dimensional (3D) diffraction data were collected as rocking curves of the rotation angle around the normal of the sample (here, $\mu$-angle of the diffractometer). Note that we normalised the data with the electron beam current in the ring. Measurements have been performed, when the sample was at $400\,^\circ \hbox {C}$ in a Ar-based gas flowed at 50 ml/min and at a pressure of 500 mbar. We do not expect dynamical structural evolution at this condition.

### Phase retrieval

The reconstruction of the Bragg electron density and phase from step-by-step and continuous BCDI were obtained using the PyNX package^32^. Phase retrieval was carried out on the raw diffracted intensity data. Defective pixels for experimental data, and gaps in the detector were masked using the bcdi package^33^, and let free during phase retrieval. The initial support, which is the constraint in direct space, was estimated from the auto-correlation of the diffraction intensity. For the data measured by continuous scanning, we applied a binning factor of 10 along the rocking direction to match the size of the step-by-step data. A series of 1400 Relaxed Averaged Alternating Reflections (RAAR^34^) plus 300 Error-Reduction (ER^35,36^) steps, including shrink wrap algorithm every 20 iterations^5^, were used. A support was built from the best reconstructed object. Afterwards, the support was fixed during the first 20 iterations and then let free, while reapplying a series of 1400 Relaxed Averaged Alternating Reflections plus 300 Error-Reduction steps, the shrink wrap algorithm was applied every 20 iterations. The phasing process included a partial coherence algorithm to account for the partially incoherent incoming wave front^37^. Note that it is important to consider partial coherence as it has been demonstrated that continuous scanning can induce a degradation in coherence^17^. We have observed that the reconstructed amplitude/modulus of the object is less homogeneous when partial coherence is not taking into account during phase retrieval (see Figure S7). To ensure the best reconstruction possible, we kept only the best 50 reconstructions (with lowest free Log-Likelihood^24^) from 500 with random phase start and performed the decomposition into modes^24^. The reconstruction was then corrected for refraction and absorption using the bcdi package^33^. After removing the phase ramp and phase offset, the data was finally interpolated onto an orthogonal grid for ease of visualisation.

## Supplementary information

Supplementary information.

## Data Availability

The data reported in this paper is available upon request. When published the data will be uploaded to the CXI database. The pre- and post-processing scripts belong to the BCDI package (https://doi.org/10.5281/zenodo.3632471), that can be downloaded from PyPI (https://pypi.org/project/bcdi/) or GitHub (https://github.com/carnisj/bcdi). The phasing algorithm PyNX is available at http://ftp.esrf.fr/pub/scisoft/PyNX/.
