# Author Correction: Continuous scanning for Bragg coherent X-ray imaging

**DOI:** 10.1038/s41598-020-75649-7

**Published:** 2020-11-05

**Authors:** Ni Li, Maxime Dupraz, Longfei Wu, Steven J. Leake, Andrea Resta, Jérôme Carnis, Stéphane Labat, Ehud Almog, Eugen Rabkin, Vincent Favre-Nicolin, Frédéric-Emmanuel Picca, Felisa Berenguer, Rim van de Poll, Jan P. Hofmann, Alina Vlad, Olivier Thomas, Yves Garreau, Alessandro Coati, Marie-Ingrid Richard

**Affiliations:** 1 CEA Grenoble, IRIG, MEM, NRS, Univ. Grenoble Alpes, 17 rue des Martyrs, 38000 Grenoble, France; 2 ESRF - The European Synchrotron, 71 Avenue des Martyrs, 38000 Grenoble, France; 3 CNRS, Université de Toulon, IM2NP UMR 7334, Aix Marseille Université, 13397 Marseille, France; 4 Synchrotron SOLEIL, L’Orme des Merisiers, Saint-Aubin, BP48, 91192 Gif-sur-Yvette, France; 5 Deutsches Elektronen-Synchrotron (DESY), Notkestraße 85, 22607 Hamburg, Germany; 6 Department of Materials Science and Engineering, Technion-Israel Institute of Technology, 3200003 Haifa, Israel; 7 Laboratory for Inorganic Materials and Catalysis, Department of Chemical Engineering and Chemistry, Eindhoven University of Technology, P.O. Box 513, 5600 MB Eindhoven, The Netherlands; 8 Laboratoire Matériaux Et Phénomènes Quantiques, CNRS, UMR 7162, Université de Paris, 75013 Paris, France

Correction to: *Scientific Reports* 10.1038/s41598-020-69678-5, published online 29 July 2020

This Article contains errors.

In Figure 1, the numerical labels for the Log(Intensity) colour scale are in the incorrect position. The correct Figure 1 appears below.

**Figure 1:**
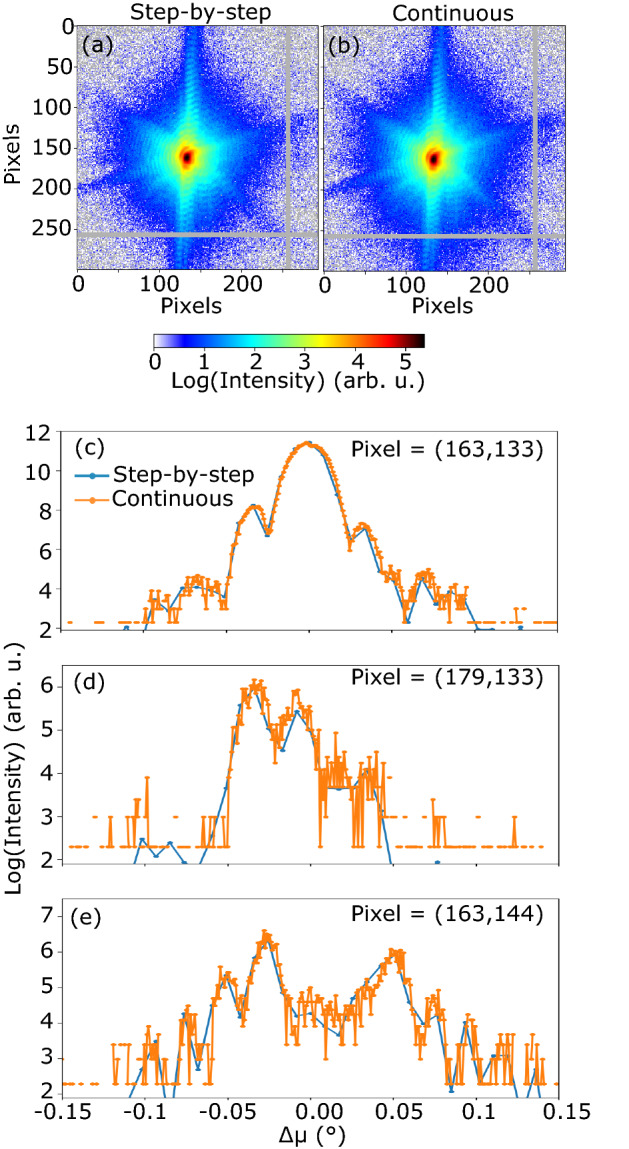
A correct version of the original Figure 1.

In Figure 2, three erroneous numerical labels ‘-3.89′, ‘0′ and ‘3.34′ appear above the Strain colour scale. The correct Figure 2 appears below.

**Figure 2:**
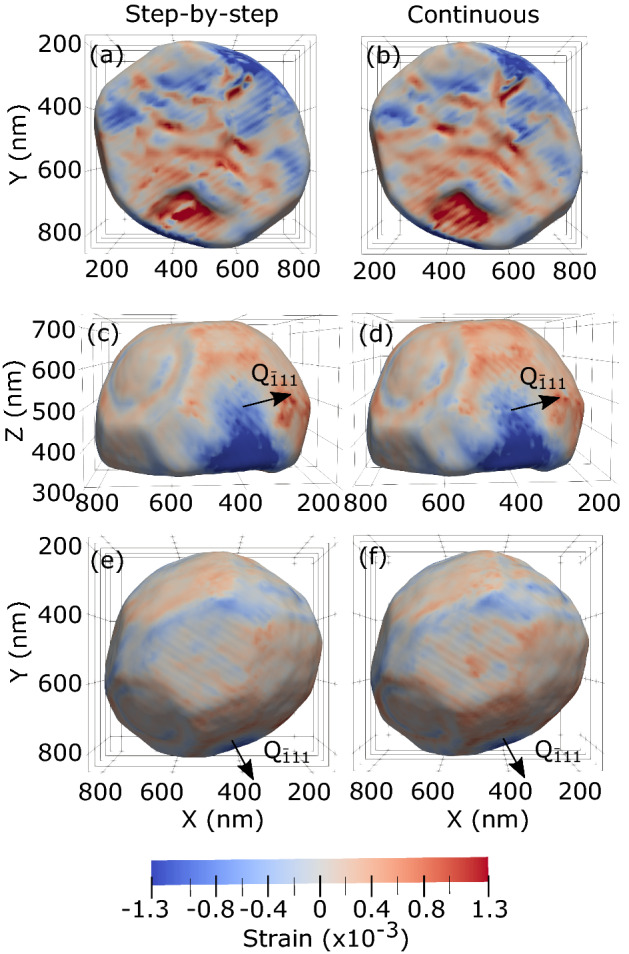
A correct version of the original Figure 2.

