# Supplementary material for: Continuous scanning for Bragg coherent X-ray imaging
Source: Sci Rep. 2020 Jul 29;10:12760. doi: 10.1038/s41598-020-69678-5 (PMC7391662; doi:10.1038/s41598-020-69678-5)
Supplement: Supplementary file 1 — Supplementary information. [file 41598_2020_69678_MOESM1_ESM.pdf]

Supplementary Materials: Continuous scanning for Bragg coherent x-ray imaging

Ni Li,[1,2] Maxime Dupraz,[1,2] Longfei Wu,[3,2] Steven J. Leake,[2] Andrea Resta,[4] Jérôme Carnis,[5] Stéphane Labat,[3] Ehud Almog,[6] Eugen Rabkin,[6] Vincent Favre-Nicolin,[2] Frédéric-Emmanuel Picca,[4] Felisa Berenguer,[4] Rim van de Poll,[7] Jan P. Hofmann,[7] Alina Vlad,[4] Olivier Thomas,[3] Yves Garreau,[4,8] Alessandro Coati,[4] and Marie-Ingrid Richard[1,2]

[1)] *Univ. Grenoble Alpes, CEA Grenoble, IRIG, MEM, NRS, 17 rue des Martyrs 38000 Grenoble, France*

[2)] *ESRF - The European Synchrotron, 71 Avenue des Martyrs, Grenoble 38000, France*

[3)] *Aix Marseille Université, CNRS, Université de Toulon, IM2NP UMR 7334, 13397 Marseille, France*

[4)] *Synchrotron SOLEIL, L'Orme des Merisiers, Saint-Aubin, BP48, 91192 Gif-sur-Yvette, France*

[5)] *Deutsches Elektronen-Synchrotron (DESY), D-22607 Hamburg, Germany*

[6)] *Department of Materials Science and Engineering, Technion-Israel Institute of Technology, 3200003, Haifa, Israel*

[7)] *Laboratory for Inorganic Materials and Catalysis, Department of Chemical Engineering and Chemistry, Eindhoven University of Technology, P. O. Box 513, 5600 MB Eindhoven, The Netherlands*

[8)] *Université de Paris, Laboratoire Matériaux et Phénomènes Quantiques, CNRS, UMR 7162, 75013 Paris, France*

(Dated: 8 July 2020)

I. GEOMETRY OF THE EXPERIMENT

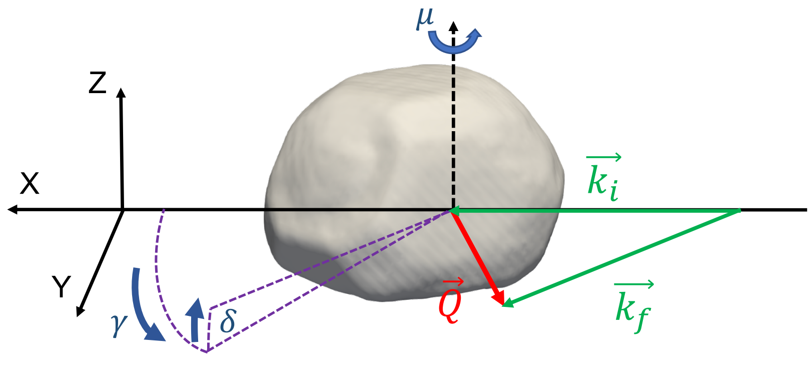

FIG. S1. Geometry of the experiment. The different angles used during the measurement are indicated as well as the wave-vectors of the incident $\mathbf{k_i}$ and scattered $\mathbf{k_f}$ x-rays. $\mathbf{k_i}$ is along the X-direction. $\mathbf{Q}$ is the scattering vector: $\mathbf{Q} = \mathbf{k_f} - \mathbf{k_i}$.

II. COMPARISON OF THE RECONSTRUCTED PHASE AND DISPLACEMENT FIELD

Figure S2 displays different views of the BCDI reconstruction of the phase and displacement field, $u_{\bar{1}11}$, drawn at 30% of the maximum Bragg electron density of the Pt nanoparticle measured by both scanning methods. As shown in Figure S2, the reconstructed phase and displacement field are essentially the same for step-by-step and continuous scanning modes. For the displacement field ($u_{\bar{1}11}$), red (positive) indicates projected displacement along the $[\bar{1}11]$ (see the arrows in Figure S2), whereas blue (negative) indicates opposite displacement. In both cases, edges of the crystal display negative displacement, while facets show preferentially positive displacement.

Figure S3 displays the central slice of the reconstructed phase in yz and xy planes for the step-by-step (a-b) and continuous (c-d) scans. As shown by the weak values of the difference of the reconstructed phases (see Figures S3 (e-f)), the phase retrieved by the two methods are in very good agreement.

Histograms of the displacement $u_{\bar{1}11}$ for both methods are displayed in Figure S3(g). The two histograms are almost superimposed, indicating that both reconstructions are in rather good agreement. We have also quantified the difference of the displacement reconstructed for both scanning

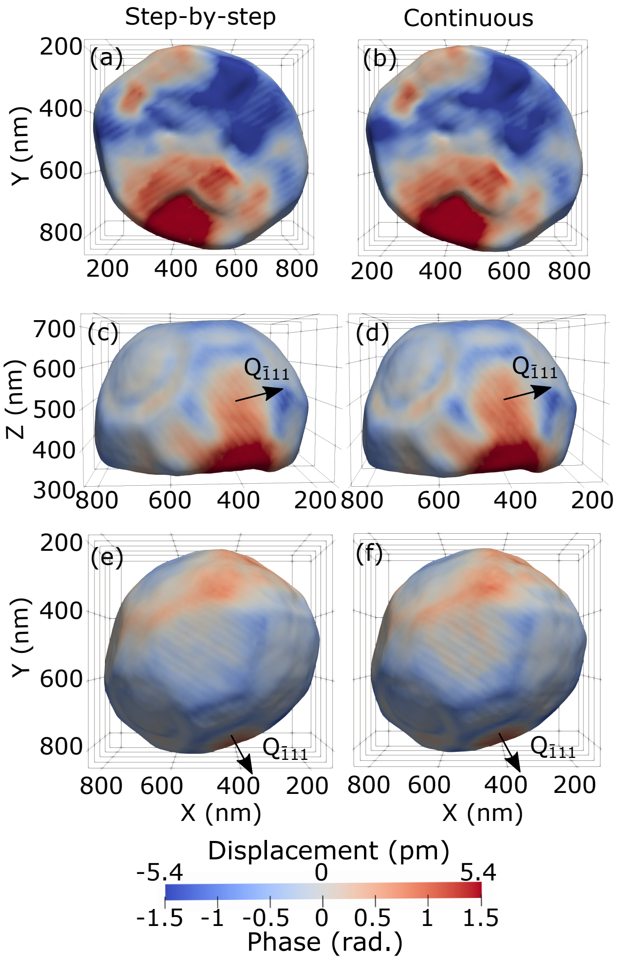

FIG. S2. Bottom (a, b), side (c, d) and top (e, f) views of the BCDI reconstruction of the phase and displacement field along the [$\bar{1}$11] direction, $u_{\bar{1}11}$, drawn at 30% of the maximum Bragg electron density of the Pt nanoparticle measured by conventional step-by-step (a, c and e) and continuous (b, d and f) scanning. The direction of the scattering vector, $\mathbf{Q}_{\bar{1}11}$, is indicated in the figure.

methods on a voxel-by-voxel basis, the voxel size being equal to 9.76^3 nm^3 for both reconstructions. The histogram shown in Figure S3(h) illustrates the dispersion of the differences in the reconstructed phase values and follows a Gaussian behaviour. For the dispersion of the differences in the reconstructed phase values, the standard deviation is 7.18×10^{-2} rad, corresponding to a small displacement of 2.6 pm, very close to the accuracy of the technique of 1 pm (the value from literature being better as multiple reflections have been used)[1].

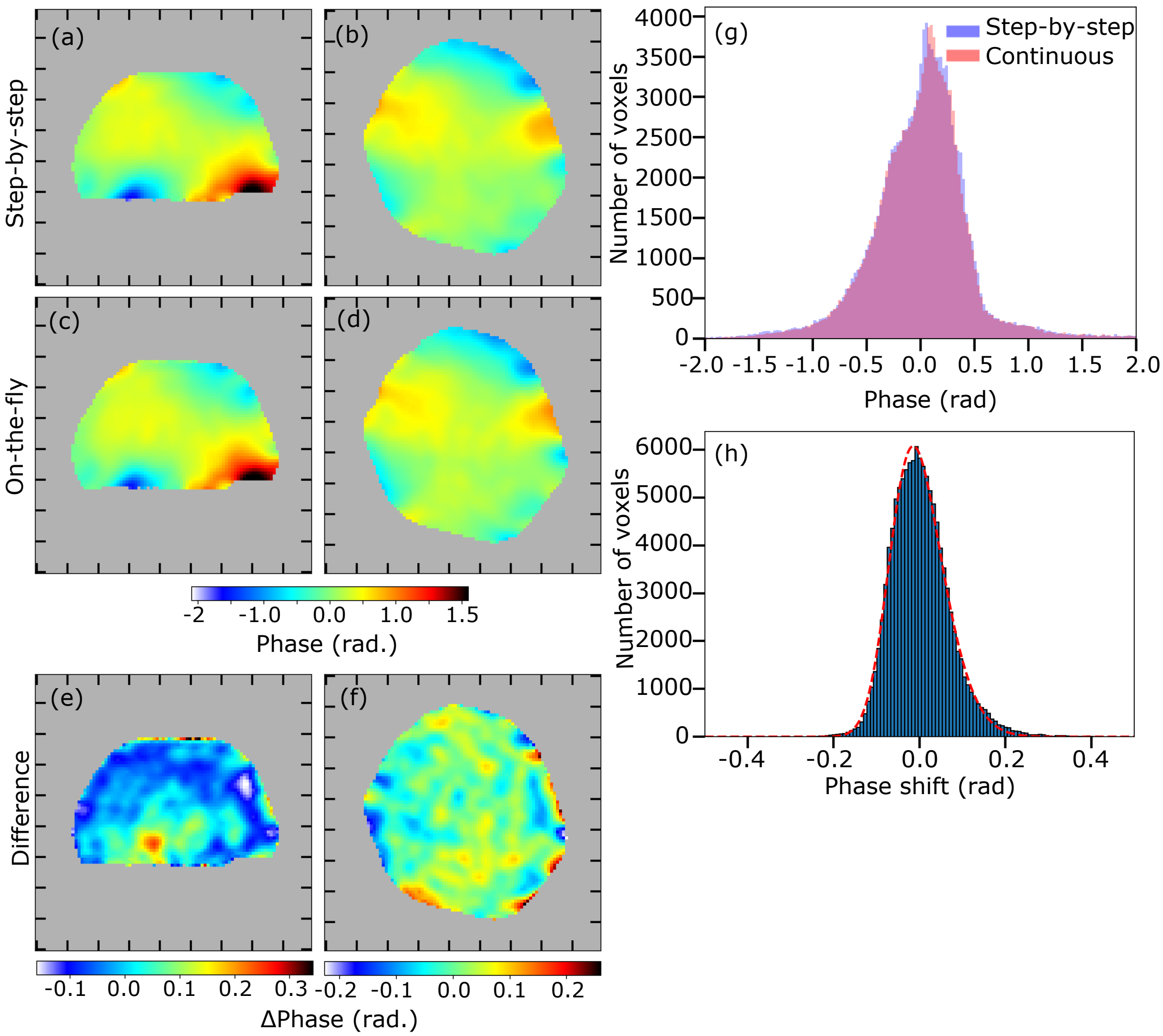

FIG. S3. (a-b): Central slice of the reconstructed phase in yz and xy plane for the step-by-step scan, drawn at 30% of the reconstructed modulus. (c-d): Central slice of the reconstructed phase in yz and xy planes for the continuous scan, drawn at 30% of the reconstructed modulus. (e-f): Central slice of the difference of the reconstructed phases in yz and xy plane, drawn at 30% of the reconstructed modulus of the step-by-step scan. Ticks correspond to 100 nm. The colorbar in the difference maps reflects the full range of the slice only, not of the full crystal. (g) Histograms of $[\bar{1}11]$ displacement ($u_{\bar{1}11}$) reconstructed for both scanning methods. (h) Histogram of the phase difference between the two scanning methods on a voxel-by-voxel basis as well as its Gaussian fit.

III. PHASE RETRIEVAL TRANSFER FUNCTION

We performed 500 independent reconstructions for both step-by-step and continuous data. The free log-likelihood indicator has been used as a metric evaluation of the solutions[2]. Figure S4(a) displays the evolution of the free log-likelihood metric sorted in an ascending order with respect to the 500 reconstructions. The free log-likelihood values are higher for the reconstructed continuous data. Figures S4(b-e) show that the reconstruction with a high free log-likelihood metric should be discarded as the shape of the crystal is no more reconstructed and that the free log-likelihood allows to discriminate between the solutions. We analysed the solutions through an eigen-decomposition. Figure S5 displays the phase retrieval transfer function (PRTF) as a function of the number of solutions (with lowest free log-likelihood) used for the eigenvalue decomposition.

We have tested the impact of binning along the rocking direction on the spatial resolution of the reconstructed crystal measured by continuous BCDI. Figure S6 estimates the spatial resolution using the phase retrieval transfer function (PRTF) for different binning factors along the rocking direction (from 1, *i.e.* no binning, to 20) of the continuous measured data. All the curves have been obtained from the first eigen-solution obtained by computing an eigenvalue decomposition over 50 best reconstructions out of 500.

IV. PARTIAL COHERENCE

Figure S7 displays the impact of partial coherence on the retrieved modulus for the continuous scan. If partial coherence is not taking into account during phase retrieval, the histogram of the retrieved modulus does not show a sharp distribution; the retrieved modulus appears less homogeneous.

REFERENCES

[1]S. Labat, M.-I. Richard, M. Dupraz, M. Gailhanou, G. Beutier, M. Verdier, F. Mastropietro, T. W. Cornelius, T. U. Schülli, J. Eymery, and O. Thomas, “Inversion Domain Boundaries in GaN Wires Revealed by Coherent Bragg Imaging,” ACS Nano **9**, 9210–9216 (2015).

[2]V. Favre-Nicolin, S. Leake, and Y. Chushkin, “Free log-likelihood as an unbiased metric for coherent diffraction imaging,” Scientific Reports **10**, 2664 (2020).

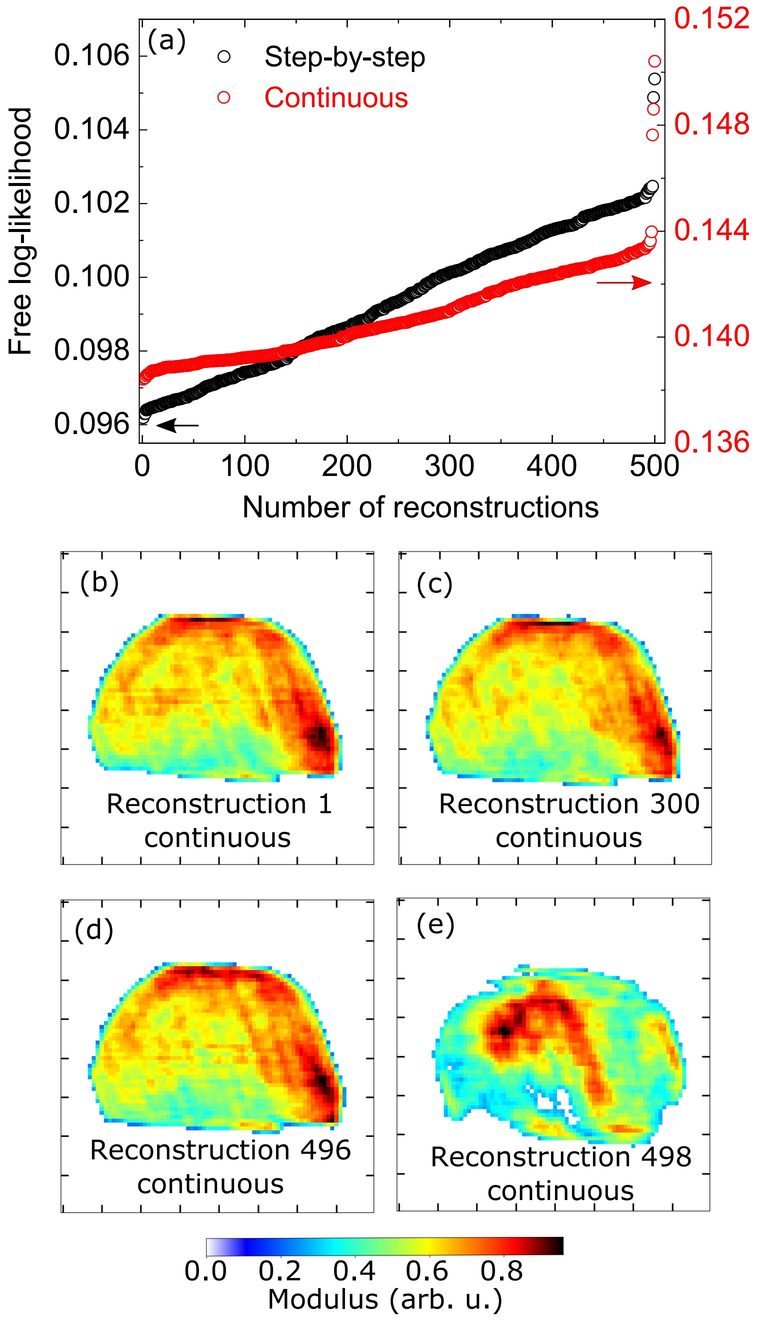

FIG. S4. (a) Evolution of the free log-likelihood metric sorted in an ascending order with respect to the number of reconstructions. We performed 500 reconstructions leading to 500 solutions. Some of the solutions, corresponding to the central slice of the reconstructed modulus in the detector plane, are displayed in Figures (b-e).

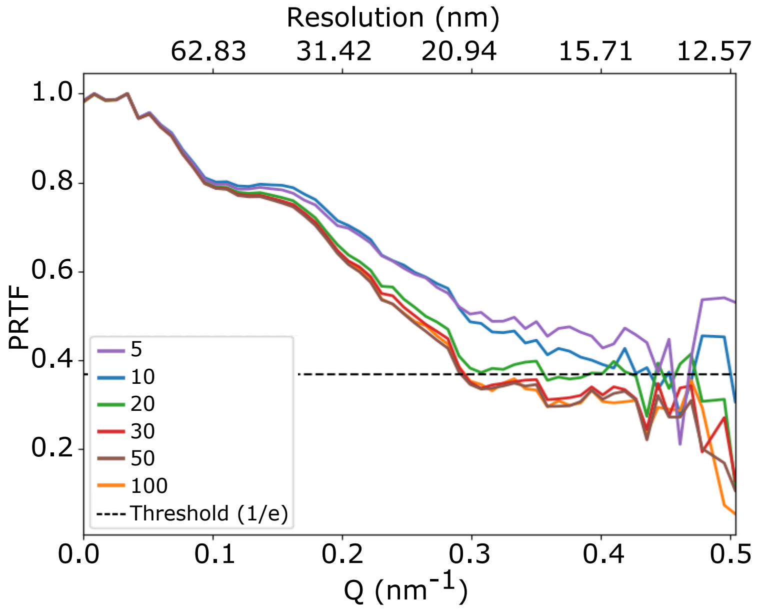

FIG. S5. Evolution of the phase retrieval transfer function (PRTF) as a function of the number of solutions (from 5 to 100) used for the mode decomposition.

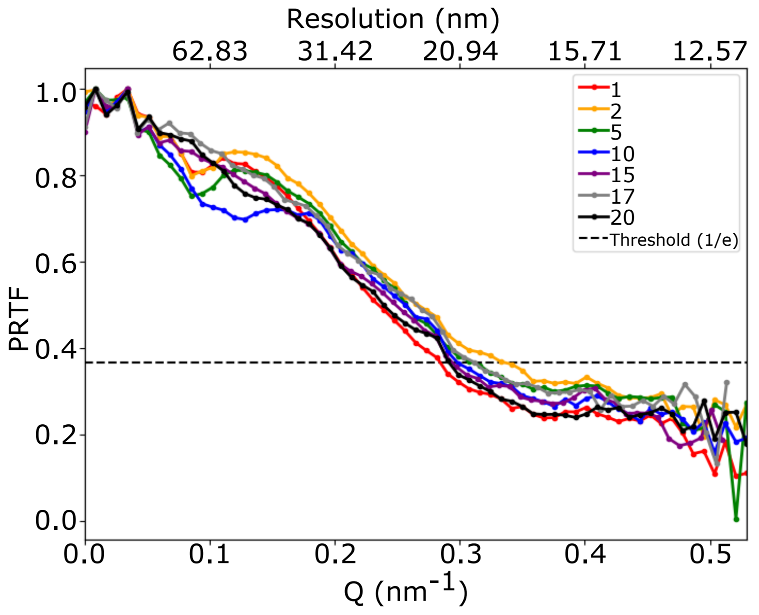

FIG. S6. Estimation of the spatial resolution using phase retrieval transfer function for different binning factors along the rocking direction (from 1, *i.e.* no binning, to 20) of the continuous measured data.

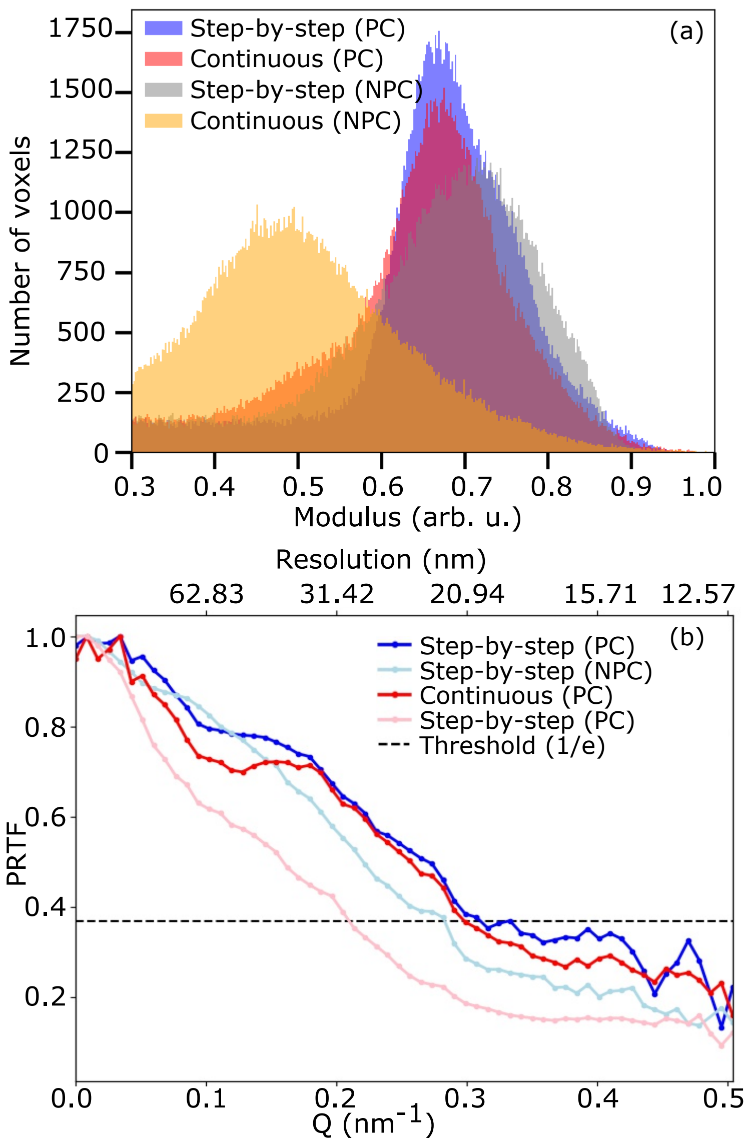

FIG. S7. (a) Histograms of the reconstructed modulus and (b) phase retrieval transfer function for the step-by-step and continuous scanning modes with (PC) and without (NPC) taking into account the partial coherence during phase retrieval.
